# Demonstration of speckle resistance using space–time light sheets

**DOI:** 10.1038/s41598-022-18153-4

**Published:** 2022-08-18

**Authors:** Mbaye Diouf, Zixi Lin, Mitchell Harling, Kimani C. Toussaint,

**Affiliations:** PROBE Lab, School of Engineering, Brown University, Providence, RI 02912 USA

**Keywords:** Nonlinear optics, Ultrafast photonics

## Abstract

The capacity of self-healing fields to reconstruct after passing through scattering media may prove useful in reducing speckle formation. Here, we study the speckle response of the space–time (ST) light sheet compared to a Gaussian wave packet, Airy beam, and Bessel Gauss beam. We find that the Pearson’s correlation coefficient for the ST light sheet is 50%, 48% and 40% larger than that of the Gaussian, Airy beam and Bessel Gauss beams, respectively, demonstrating a strong correlation to an input beam that has not been speckled. These results suggest that the ST light sheet exhibits considerable resistance to speckle generation. We also investigate the speckle response of the ST light sheet at its second-harmonic frequency and observe a mean Pearson’s correlation coefficient close to 0.6, comparable to the second-harmonic Bessel Gauss beam, and 2.8 × the value obtained for the second-harmonic Gaussian beam. Our results lend themselves to a variety of applications including bioimaging, communications, and optical tweezers.

## Introduction

Speckle is an optical interference phenomenon that occurs when a coherent field interacts with materials having rough features on the scale of an optical wavelength^1^. Speckle can appear as random granularity in images, obscuring valuable data. This granularity often manifests in ultrasound and optical coherence tomography, making speckle reduction a topic of interest in medicine^2,3^ as well as microscopy^4,5^ and encryption^6^. Upon encountering a surface with wavelength-order roughness, coherent light experiences many independent scattering events. Thus, the scattered components gain unique and random phase delays; speckle noise is the result of these scattered components interfering in the imaging plane or at a sensor^1,7^.

Various techniques have been introduced to reduce speckle including soft computing approaches (e.g., neural networks)^8^, rotating diffusers to decrease the spatial coherence of lasers^5^ and substituting partially coherent or incoherent light sources for lasers. Incoherent light sources are often the solution.

However, compared to lasers, they lack the favorable attributes of beams such as the ability to be tightly focused and concentrated in optical power, which permit, for example, the generation of nonlinear optical phenomena. Adapting a readily available coherent laser to be speckle resistant is therefore desirable for many research applications. Near-diffraction-free, self-healing optical fields are another potential solution to the speckle problem that can be generated using extra cavity optics such as a spatial light modulator (SLM). A cheaper option includes employing an axicon lens to generate near-diffraction-free Bessel beams.

The ideal Bessel beam is a monochromatic field that does not undergo diffractive spreading upon free propagation^9,10^. However, such a beam cannot be physically realized, and thus often the near-diffraction-free Bessel-Gauss (BG) beam is used as it approximates the characteristics of a Bessel beam. It has been demonstrated that BG beams can be used to control the size of speckles and generate non-diffractive speckle patterns^11^. Both Airy and BG beams also have a self-healing^12,13^ property, which has been demonstrated in microscopy and quantum optics applications^14,15^. BG beams have also been shown to produce self-reconfiguring speckle patterns^16^. To the best of our knowledge, BG beams have not been explored for their potential to reduce speckle in imaging. However, the unique control that the BG beam has demonstrated over speckle and its self-healing through scattering media has motivated us to investigate the potential resistance to speckle for space–time (ST) light sheets.

The ST light sheet or ST wave packet is a classically entangled field exploiting unique correlations between spatial frequencies and temporal frequencies to achieve diffraction-free and dispersion-free properties^17–19^. Furthermore, because of the tight spatiotemporal correlations, the group velocity in free space can be precisely controlled to be subluminal or superluminal^20^. These one-dimensional ST light sheets have demonstrated self-healing similar to a BG beam^21–23^. Previously reported spatiotemporal orbital angular momentum of light^24,25^ that have circularly symmetric intensity profiles, but do not show self-healing, should not be confused with the ST light sheet studied here.

In this work, we investigate the propagation of the ST light sheet after its interactions with several scattering media: a thin holographic diffuser, fluorescent beads, and tendon sample, and compare the resistance to speckle generation to that of a Gaussian wave packet, a BG beam, and a 1D Airy beam at different propagation planes. We investigate the speckle response of the Gaussian beam, BG beam and the ST light sheet at the second-harmonic (SH) frequency. We demonstrate that the generated SH ST-like light sheet is resistant to the development of speckles, making it a suitable illumination source for imaging with a mean Pearson's correlation coefficient of 0.56. Conversely, the corresponding SH Gaussian beam and BG beam have a mean coefficient correlation of 0.20 and 0.55, respectively. To the best of our knowledge, the ability of ST light sheets to resist speckle generation in scattering media has not been examined.

## Experiment

**Figure 1:**
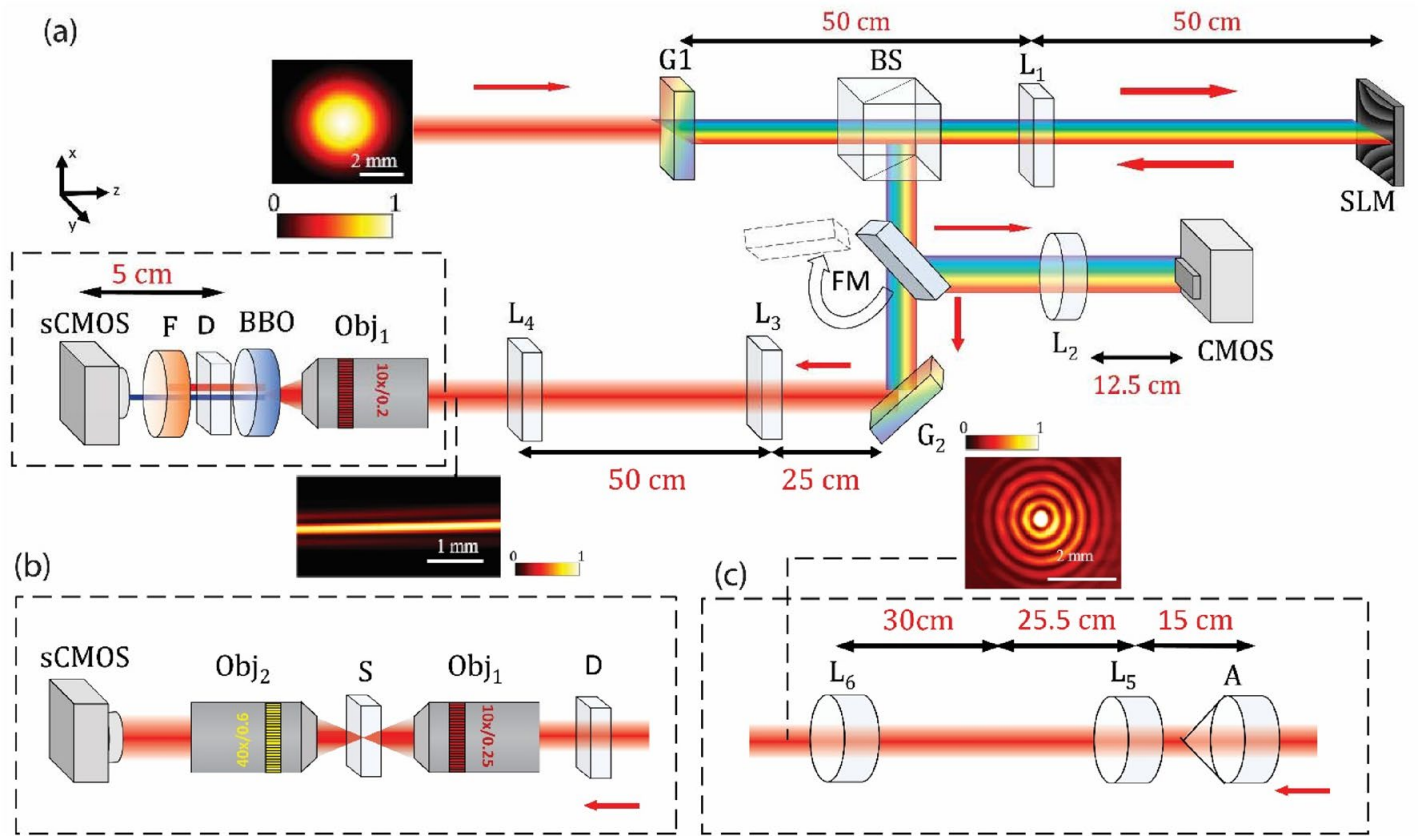
Experimental setup for the generation of the ST light sheet and SH ST-like light sheet (**a**). Schematic of the imaging system post-diffuser using fluorescent beads and tendon as samples (**b**). Generation of the BG beam via an axicon lens (**c**). Insets show the intensity distributions of the Gaussian beam, ST light sheet, and the BG beam. For the optical components: G_1_ and G_2_: diffraction grating with 1200 grooves/mm (G_1_ is used in reflection mode but is depicted here in transmission mode for simplicity), BS: Beam splitter, SLM: spatial light modulator, L_1_, L_3_, and L_4_: Cylindrical lens, D: holographic diffuser 0.78 mm thickness, Obj: objective, S: sample, BBO: nonlinear crystals β-barium borate 1 mm thickness. F: second-harmonic generation filter and sCMOS, camera, L_2_, L_5_, and L_6_: spherical lens. The red arrows indicate the beam propagation direction.

The experimental setup used in this work is depicted in Fig. 1a. We operate a horizontally polarized ultrafast laser at 800 nm (InSight X3, Spectra Physics; bandwidth of ~ 8.5 nm, pulse repetition rate of ~ 80 MHz). The size of the beam is expanded to 25 mm before the pulse spectrum is spatially dispersed along the horizontal axis by the grating, G_1_. Via a cylindrical lens, L_1_ (focal length 500 mm), the first diffraction order of the pulse is directed to a polarization-dependent SLM (Hamamatsu, X15213-07). The SLM displays a 2D phase pattern that assigns a prescribed spatial frequency to each wavelength, converting the field to a ST light sheet. The 2D phase pattern is generated using a spectral tilt angle of 44.98°. The wave-packet group velocity is determined by the spectral tilt angle $v_{g} = {\text{c tan}}\theta$ where c is the speed of light and $\theta$ is the spectral tilt angle.

The beam is then directed to spherical lens L_2_ (focal length 125 mm) through a flip mirror (FM) between the beam splitter, BS and grating G_2_, resulting in the spatio-temporal spectrum of the ST light sheet at the CMOS, as shown in Fig. 1a. The spatio-temporal spectrum of the ST light sheet is shown in Fig. S1 in the supplemental material. The wave packet is reflected back through L_1_ and then redirected to the second grating, G_2_, by the BS, whereby the ST light sheet is formed. Cylindrical lenses L_3_ (focal length 400 mm) and L_4_ (focal length 100 mm) are used to demagnify the ST light sheet. The transverse intensity profile of the ST light sheet is shown in the inset of Fig. 1 (after L_4_). The width (along the transverse direction) of the ST light sheet is ~ 90 µm. We use a 0.78-mm thick holographic diffuser (Edmund Optics, 47-993) with dimensional tolerance of 0.5 mm to explore the ST light sheet’s resistance to speckle generation in comparison to that experienced by a standard Gaussian wave packet, and BG beam. See the insets of Fig. 1 for the transverse intensity profiles of these fields. We also compare the speckle resistance of the ST light sheet and the Airy beam, note that, we generate the Airy beam by applying the appropriate phase pattern on the SLM (not shown here).

We add a 10 ×, 0.25 NA objective lens, Obj_1_, (Olympus America, ACHN 10XP) to focus the fields onto a β-Barium Borate (BBO) crystal of 1-mm thickness (EKSMA Optics, BBO-604H). We position the diffuser to be 5 cm from the camera for all SH beams in order to assess the speckle response with propagation for all three beams. The SH signals are isolated using a narrow bandpass filter, F, (Semrock, FF01-390/18-25) and then collected using a sCMOS camera. In our study, we used an average power of 1.5 mW to generate the SH of each field. Note that this average power is a constraint placed by the relative low throughput associated with generating the ST light sheet. This is a direct result of the combined effects of the beam splitter, gratings, and SLM. This also constrains where we place D for our SH experiments.

Figure 1b details the imaging system used in this experiment. Here, the sample, S, corresponds to one of the following: 1-µm diameter fluorescent beads (Thermo Scientific, F8819) suspended in 1 mL of agarose solution, or 200-µm thick cow tendon prepared via cryostat (Thermo Scientific, Cryostat NX50). A cow-tendon tissue microarray sample is used. The samples are mounted on a glass slide and microscope coverslip. For the illumination we use objective (Obj_1_). Forward propagating signals are collected using a 0.6 NA 40 × objective (Obj_2_) lens (Nikon, CFI S Plan Fluor ELWD 40XC). Figure 1c shows the setup for generation and subsequent collimation of a zero-order BG beam using, respectively, an axicon lens (Thorlabs, AX2505-B) and two spherical lenses, L_5_ (focal length 25.5 mm) and L_6_ (focal length 300 mm)^26^.

## Results and discussion

**Figure 2:**
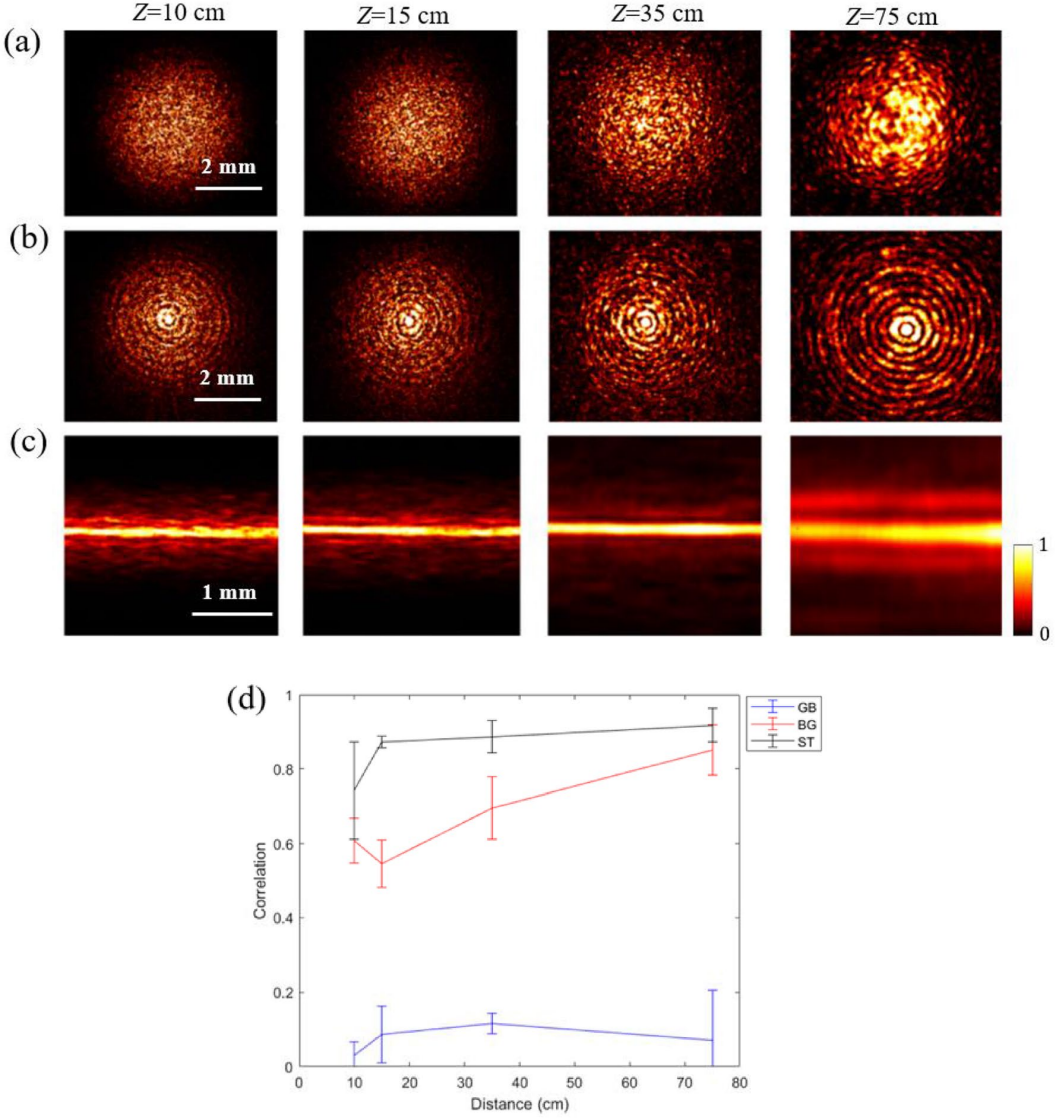
The Intensity distributions at different propagation planes (10, 15, 35, and 75 cm) upon transmission through diffuser D for illumination by a Gaussian wave packet (**a**), BG beam (**b**), and ST light sheet (**c**). The PCC at different propagation distances upon transmission through diffuser D and without diffuser for illumination by a Gaussian beam GB (blue), BG beam (red) and ST light sheet (black) (**d**).

Figure 2a–c depicts the intensity distributions for the Gaussian beam, BG beam, and ST light sheet, respectively, after passing through the holographic diffuser, at propagation distances of 10, 15, 35, and 75 cm. The mean value of the speckle size is about 30 μm at a propagation distance of 10 cm. The speckle size varies as the propagation distance increases. The Gaussian and BG beams show speckle at all propagation distances, as seen in Fig. 2a,b, however, the ST light sheet shows progressively less speckle with propagation, and appears to reconstruct after 35 cm, as seen in Fig. 2c. After 75 cm, the ST light sheet exhibits a significant resemblance to the unperturbed ST light sheet shown in the inset of Fig. 1. Furthermore, the diffuser appears to have little influence on the underlying structure of the ST light sheet.

While the BG beam at 75 cm exhibits a resemblance to the unperturbed BG beam shown in the inset of Fig. 1, there is still a significant speckle pattern. Qualitatively, these data suggest that the ST light sheet is most robust to speckle generation.

To quantify the resistance to speckle of the Gaussian beam, BG beam, and ST light sheet, we utilize the Pearson’s correlation coefficient (PCC) which determines the similarity between two images. For monochromatic images, the PCC *r* is defined as^27^

1 $$r = \frac{{\mathop \sum \nolimits_{{\text{i}}} {\text{(x}}_{{\text{i}}} - {\text{x}}_{{\text{m}}} {\text{)(y}}_{{\text{i}}} - {\text{y}}_{{\text{m}}} {)}}}{{\sqrt {\mathop \sum \nolimits_{{\text{i}}} {\text{(x}}_{{\text{i}}} - {\text{x}}_{{\text{m}}} {)}^{{2}} } \sqrt {\mathop \sum \nolimits_{{\text{i}}} {\text{(y}}_{{\text{i}}} - {\text{y}}_{{\text{m}}} {)}^{{2}} } }},$$

where *x*_*i*_ and *y*_*i*_ are intensity values of the *i*th pixel in the 1st and 2nd image, respectively, and *x*_*m*_ and *y*_*m*_ are the mean intensity values of the 1st and 2nd image, respectively^27^. Here *r* = 1 when the two images are totally similar, and *r* = 0, or − 1, if they are uncorrelated or anti-correlated, respectively.

We measure the degree of reconstruction by calculating the PCC for the Gaussian beam, the BG beam, and the ST light sheet with and without the presence of diffuser at each propagating distance of 10, 15, 35, and 75 cm. The mean PCC is plotted as a function of propagation distance as shown in Fig. 2d. For the Gaussian beam, the mean PCC remains below 0.07 as the beam propagates toward 75 cm, indicating extremely low similarity between the unperturbed Gaussian beam and the beam perturbed by the diffuser. For the BG beam, the mean PCC shows an increasing trend from 0.6 at 10 cm to 0.85 at 75 cm after the diffuser, which means the BG beam gradually reconstructs after being scattered by the diffuser. There is a drop in the PCC for the BG beam as it propagates from 10 to 15 cm that is due to the initial distortion in the primary intensity ring surrounding the main central intensity lobe. For the ST light sheet, the mean PCC increases from 0.74 at 10 cm to 0.91 at 75 cm after the diffuser, showing high similarity between the unperturbed and perturbed beam. The PCC for the ST light sheet increases by 1300% and 20% as compared to the Gaussian and BG beams, respectively. Overall, the effect of the diffuser remains extremely small for the ST light sheet.

**Figure 3:**
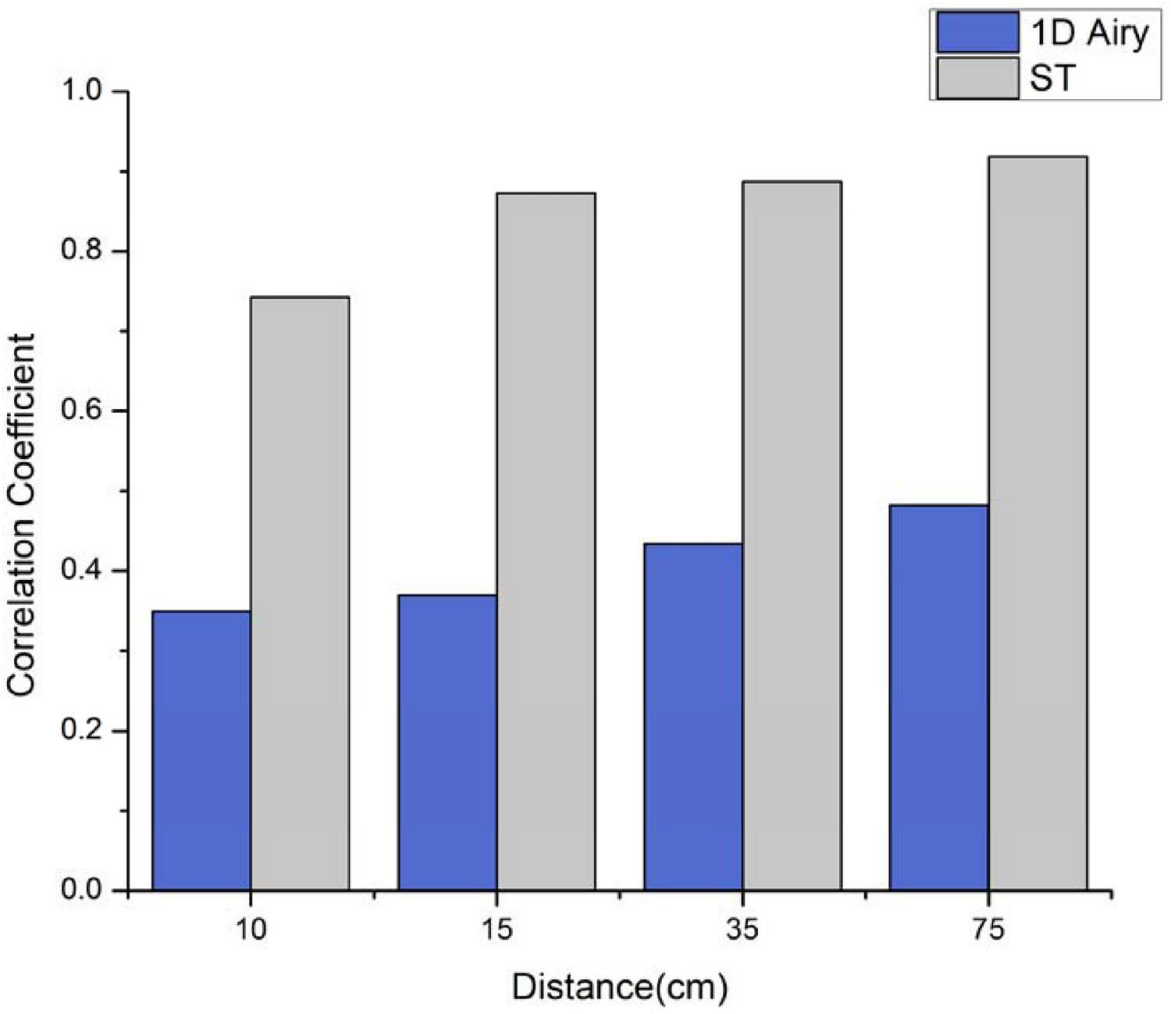
Comparison of the PCC obtained for the 1D Airy beam (blue) and ST light sheet (grey) at different propagation distances from the diffuser.

The calculated PCC for the Airy beam increases from a mean value of 0.35 at 10 cm to ~ 0.5 at 75 cm after the diffuser as observe in Fig. 3. The intensities distributions of the Airy beams are shown in Fig. S2 in the supplement document. In comparison, the mean PCC for the ST light sheet increases from 0.74 at 10 cm to 0.91 at 75 cm. Therefore, in comparison to the Airy beam, the diffuser's effect on the ST light sheet is extremely minor as shown in Fig. 3. These results suggest that the ST light sheet is more resistant to speckle generation than Airy beams.

Next, we investigate the effects on the input optical pulses when the diffuser is placed after the BBO crystal, as observed in Fig. 1, and image the resultant SH intensity profile.

**Figure 4:**
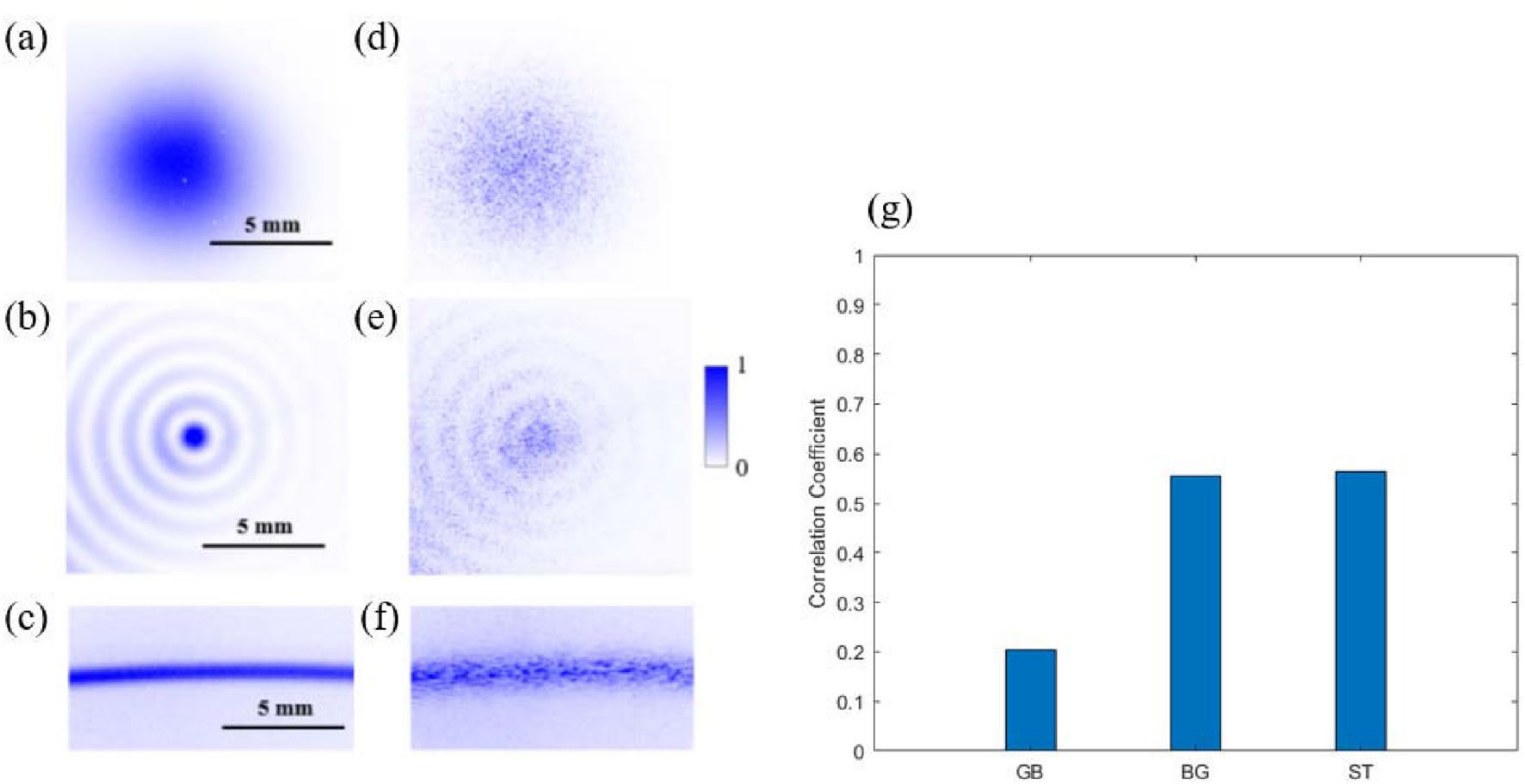
The SH intensity distribution resulting from the focused Gaussian wave packet (**a**), BG beam (**b**), and ST light sheet (**c**). The corresponding SH intensity distribution upon transmission through diffuser D of the Gaussian wave packet (**d**), BG beam (**e**), and ST light sheet (**f**). Comparison of the PCC values produced by the SH of each field after passing through the diffuser (**g**).

Figure 4a–c depicts the converted SH intensity distributions for each beam type in the absence of a diffuser. The SH signals produced by the Gaussian wave packet after passing through the diffuser are noticeably of reduced intensity compared to that of the BG beams and the ST light sheet, as observed in Fig. 4d–f. Here, we again use the mean of the PCC as a metric of quantitative comparison and find that for the Gaussian beam, BG beam, and ST light sheet *r* = 0.20, *r* = 0.55, and *r* = 0.56, respectively, see Fig. 4g. This suggests that the ST light sheet and the BG beam undergo less pulse broadening due to scattering than the Gaussian beams.

**Figure 5:**
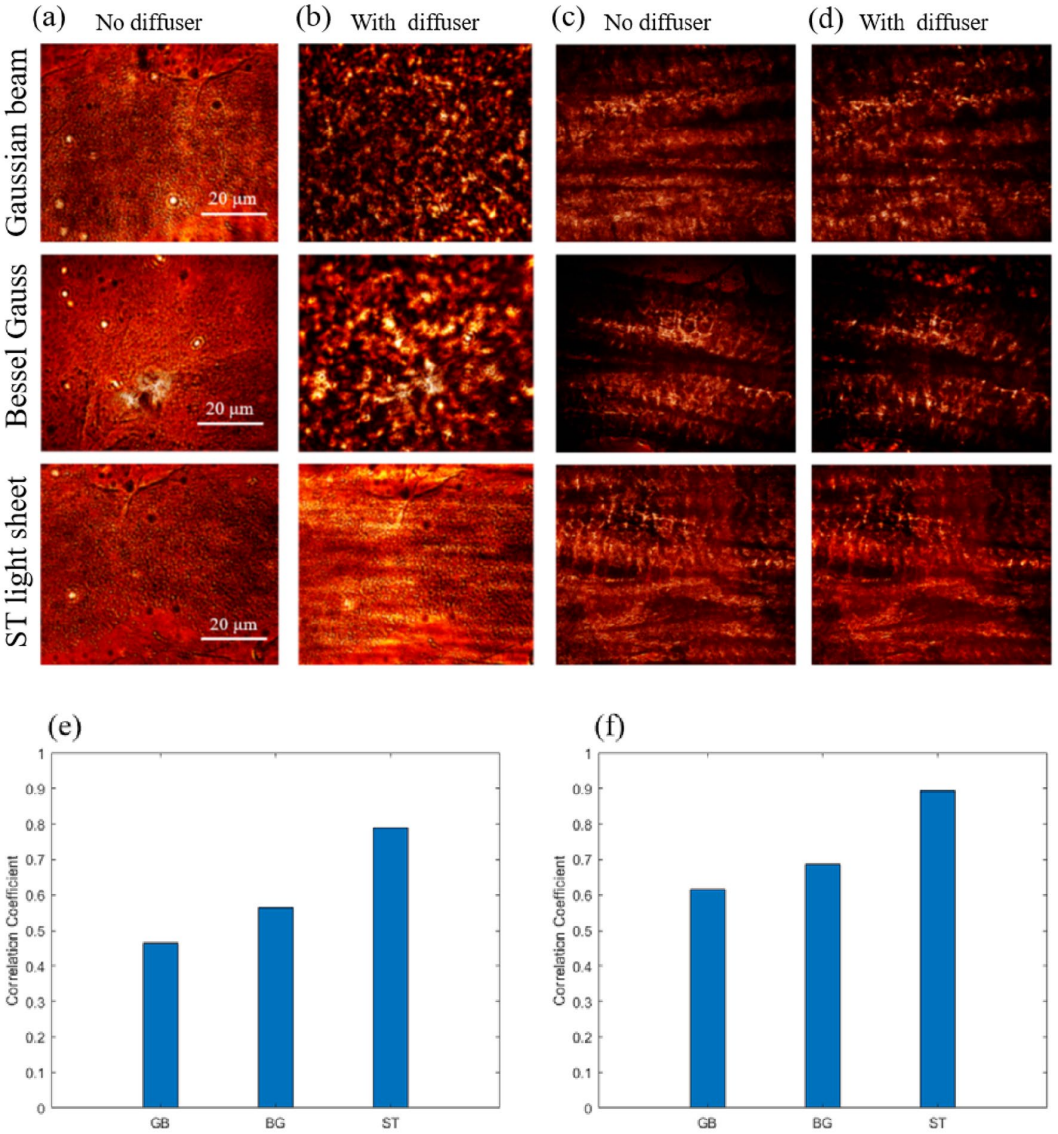
Intensity images of fluorescent beads (**a**,**b**) and tendon sample (**c**,**d**). Comparison of the correlation of the Gaussian beam, BG beam and ST light sheet for the 1-µm bead images (**e**), similar results for the tendon sample (**f**).

We next explore the response to speckle for two samples: a distribution of 1-µm diameter beads, and 200-µm cow tendon. Figure 1b shows the setup of the experiment. The first two columns of Fig. 5a,b correspond to images of the beads without and with the diffuser, respectively. Similarly, the last two columns of Fig. 5c,d correspond to images of the tendon without and with the diffuser, respectively. Qualitatively, we clearly observe the stronger speckle generation for the bead plus diffuser images for the Gaussian and BG beam as compared to the ST light sheet. The corresponding quantitative response using the mean of the PCC is shown in Fig. 5e for the beads and Fig. 5f for the tendon. We find *r* to be 0.46, 0.56, and 0.79 for the bead sample and 0.61, 0.68, and 0.90 for the tendon sample for the Gaussian beam, BG beam and ST light sheet, respectively. The *r* for the ST light sheet is 50%, and 40% larger than that of the Gaussian and BG beams, respectively. Our results suggest that the scattering operations of the diffuser have negligible influence on the ST light sheet in the context of an imaging system.

On the surface it appears that the origin of the speckle resistance could be the ‘self-healing’ effect associated with diffraction-free beams, as we observed some speckle reduction for both the Bessel Gauss and Airy beams in comparison to the Gaussian beam. However, our results also indicate that the ST light sheet outperforms these beams in this regard. This suggests that there could be a feature unique to ST light sheets perhaps arising from their tight correlation between spatial frequencies and temporal frequencies. This observation necessitates development of a full theoretical framework of ST light sheets and their propagation in turbid environments.

## Conclusion

In summary, we investigated the speckle response of the ST light sheet upon propagation through various scattering materials, such as fluorescent beads, tendon tissue, and a thin diffuser. We found that compared to the Gaussian, Airy beam and BG beams, the ST light sheet exhibits strong speckle resistance with a significantly higher PCC. We also found the SH of the ST light sheet to exhibit some resistance to speckle formation. The results of this work could be useful for a wide range of optical applications including bioimaging, communications, and optical trapping.

## Materials and methods

### Sample preparation

Cow-tendon tissue microarray samples are employed. Cow-tendon was obtained from a local abattoir and kept in the freezer. A cryostat (Thermo Scientific, Cryostat NX50) is used to slice 200 µm of tendon tissue. The fluorescent beads which diameter of 1.0 µm (Thermo Scientific, F8819) are mixed and resuspended in 1 mL of agarose solution. The samples are mounted in a mounting medium and placed on a microscope coverslip.

### Space time light sheet generation

A femtosecond laser (InSight X3, Spectra-Physics) is used to generate the ST light sheet with 150 fs pulses at a repetition rate of 80 MHz. The excitation wavelength for this work is centered at 800 nm, and the laser is spectrally adjustable between 680 and 1300 nm. For the light sheet used in the paper, we use the subluminal regime with θ = 44.98° corresponds to spectral tilt angles in the range of 0° < θ < 45°. The superluminal of ST light sheet corresponds to spectral tilt angles of 45° < θ < 180°, with a positive group velocity (vg). We measured the bandwidth of the ST light sheet ∆λ = 2 nm (see Fig. S1 in the supplemental material). These variables are essential for keeping the ST light sheet and avoiding spatial spreading.

## Supplementary Information

Supplementary Information.

## Data Availability

Relevant data that support the results of this work could be found within the paper, Supplementary Information and from the corresponding author upon request.
