# Author Correction: Demonstration of speckle resistance using space–time light sheets

**DOI:** 10.1038/s41598-022-22375-x

**Published:** 2022-10-21

**Authors:** Mbaye Diouf, Zixi Lin, Mitchell Harling, Kimani C. Toussaint

**Affiliations:** PROBE Lab, School of Engineering, Brown University, Providence, RI 02912 USA

Correction to: *Scientific Reports* https://doi.org/10.1038/s41598-022-18153-4, published online 18 August 2022

The original version of this Article contained an error in Figure 2, where Z = 75 cm was incorrectly given as Z = 35 cm in panel (a). The original Figure 2 and accompanying legend appear below.

The original Article has been corrected.

**Figure 2:**
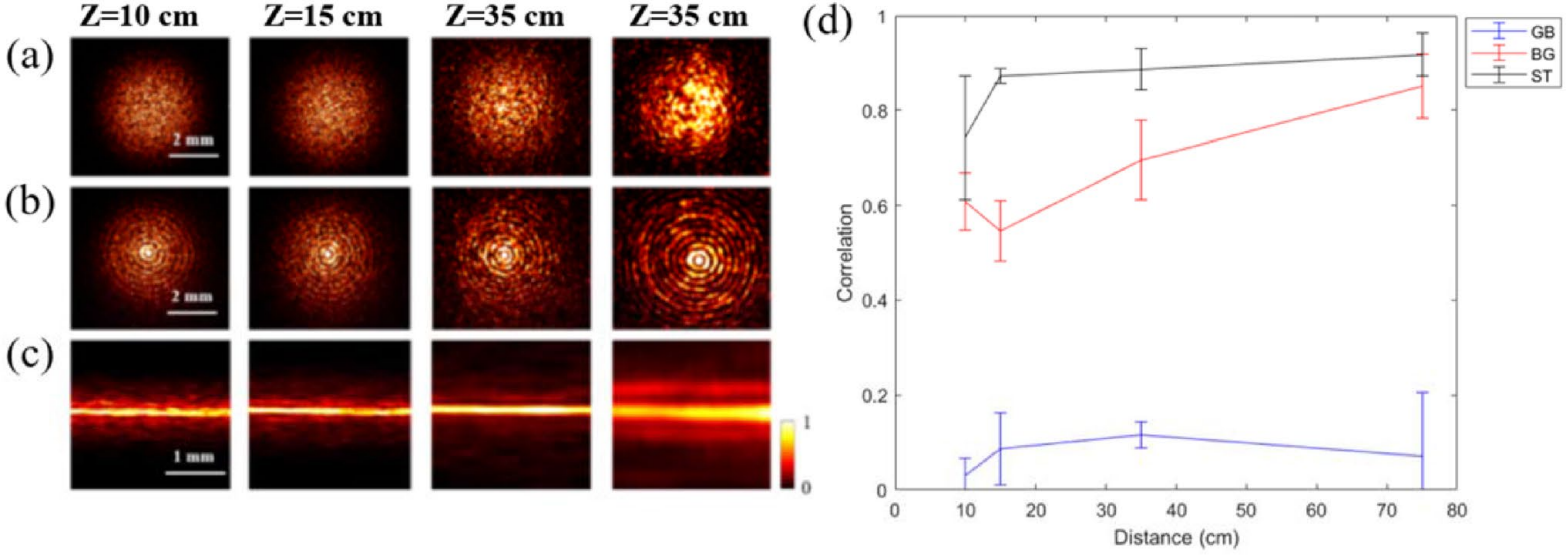
The Intensity distributions at different propagation planes (10, 15, 35, and 75 cm) upon transmission through diffuser D for illumination by a Gaussian wave packet (**a**), BG beam (**b**), and ST light sheet (**c**). The PCC at different propagation distances upon transmission through diffuser D and without diffuser for illumination by a Gaussian beam GB (blue), BG beam (red) and ST light sheet (black) (**d**).

