# Supplementary material for: Demonstration of speckle resistance using space–time light sheets
Source: Sci Rep. 2022 Aug 18;12:14064. doi: 10.1038/s41598-022-18153-4 (PMC9388688; doi:10.1038/s41598-022-18153-4)
Supplement: Supplementary file 1 — Supplementary Information. [file 41598_2022_18153_MOESM1_ESM.docx]

**Supplementary Material:**

Demonstration of speckle resistance using space-time light sheets

Mbaye Diouf, Zixi Lin, Mitchell Harling, and Kimani C. Toussaint, Jr.*

*PROBE Lab, School of Engineering, Brown University, Providence, RI 02912, USA*

[*kimani_toussaint@brown.edu](mailto:*kimani_toussaint@brown.edu)

**Theory**

The governing theory of space-time (ST) light sheets is reported in [15, 16]. Here, we use one-dimensional light sheets with an electric field *E*(*x, z*; *t*)*,* where *x* is the transverse coordinate, *z* is the axial coordinate, and *t* is time.

By appropriately designing the function $\text{ω}\text{(|}\text{k}_{\text{x}}\text{|)}$, a linear relationship between spatial and temporal frequencies, *k_x_* and *ω*, respectively, of the form $\text{ω}/{\text{c = }\text{k}_{\text{0}}\text{+}\left( \text{k}_{\text{z}}-\text{k}_{\text{0}} \right)\tan\text{θ}}$ can be established, where *k_0_* is a fixed wave number and $\text{θ}$is the spectral tilt angle, and the spatial bandwidth $\text{∆}\text{k}_{\text{x}}$ is now correlated to the temporal bandwidth $\text{∆}\text{ω}$. The ST light sheet has the form [15]:

| $\text{E}\text{(}\text{x, z}\text{;}\text{ t}\text{)=}\text{e}^{\text{i}\left. (\text{k}_{\text{0}}\text{z-}\text{ω}_{\text{0}}\text{t}\text{)} \right.}\int\text{d}\text{k}_{\text{x}}\tilde{\text{ψ}}\left. \left. {\text{(}\text{k}}_{\text{x}}\text{)} \right. \right.\text{e}^{\text{i}\left. \{\text{k}_{\text{x}}\text{x}\text{+}\left. [\text{k}_{\text{z}}\left. (\vert\text{k}_{\text{x}}\vert) \right.-\text{k}_{\text{0}} \right.\text{](}\text{z}-\text{ct}\tan\text{θ}\text{)}\text{\}} \right.}=\text{e}^{\text{i}\text{(}\text{k}_{\text{0}}\text{z}-\text{ω}_{\text{0}}\text{t}\text{)}}\text{ψ}\text{(}\text{x, z}-\text{v}_{\text{g}}\text{t}\text{)}$. | (1) |
| --- | --- |

Where *v_g_* is the group velocity along the *z*-axis, and it is given by $\text{v}_{\text{g}}\text{=}\text{c}\tan\text{θ}\text{ }.$Where *ω_0_* is the temporal frequencies respectively, and k_x_ and k_z_ are the transverse and longitudinal components of the wave vector along the x and z coordinates, respectively.

Fig. S1(a, b) depict the spatiotemporal spectrum $\left| \tilde{\text{E}}\left( \text{k}_{\text{x}}\text{, }\text{λ} \right) \right|^{\text{2}}$. The uncertainty δλ=(δ*ω*λ^2^)/2π*c*, which is smaller than the full spectrum bandwidth ∆*ω*, determines the intensity of the correlation introduced in the spatiotemporal spectrum shown in Fig. S1.


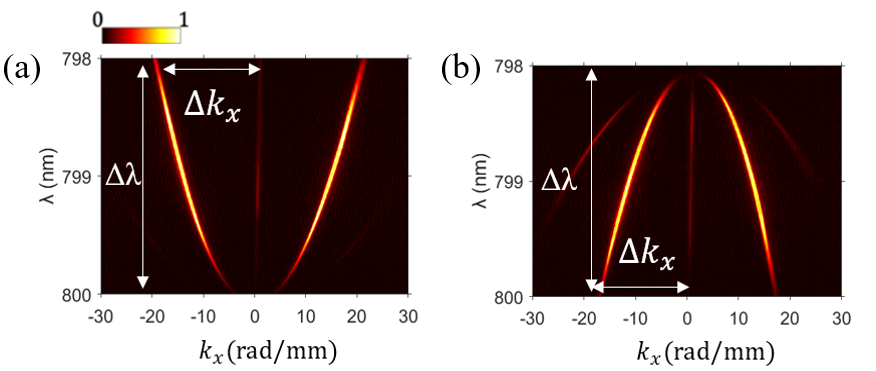


**Figure S1**. Measured spatiotemporal spectral intensity $\left| \tilde{\text{E}}\left( \text{k}_{\text{x}}\text{, }\text{λ} \right) \right|^{\text{2}}$ for subluminal (*θ* = 44.95; an ellipse) (a) and superluminal (*θ* =45.02; a hyperbola) (b) ST wave packets. Both spectra appear approximately as parabolas because of the limited bandwidth (∆*λ* ~ 2 nm).


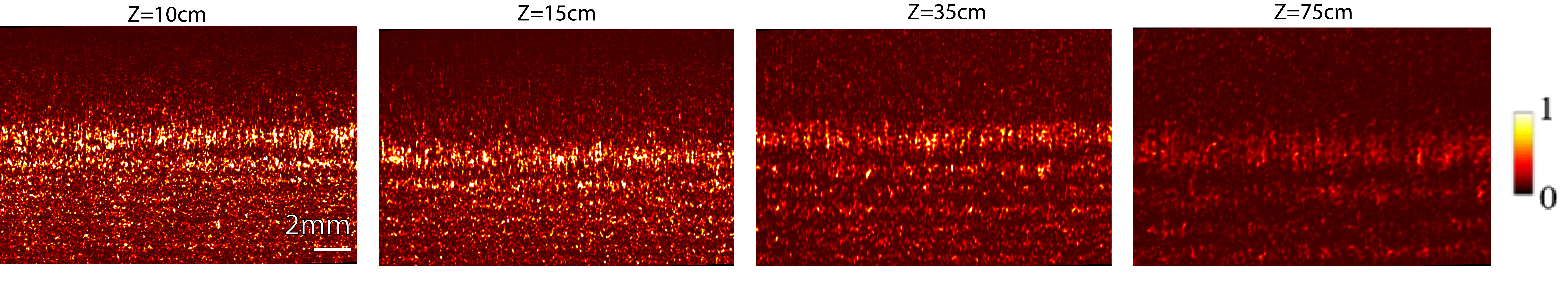


**Figure S2.** The intensity distribution of the 1D Airy beam recorded at different propagation planes (10, 15, 35, and 75 cm) upon transmission through diffuser D.

Figure S2 shows the intensity distributions of the Airy beam after passing through the diffuser for propagation distances of 10, 15, 35, and 75 cm. These results suggest that the ST light sheet is more resistant to speckle generation than Airy beams.

The near-diffraction-free behavior of the second harmonic ST light sheet was investigated for spectral tilt angles of 45.01°, 45.02°, 44.99° and 44.8°. The intensity is measured using a sCMOS for a 500-ms exposure time in each case. Figure S3(a, b) shows that the subluminal and superluminal second-harmonic ST wave packets exhibit similar structures.


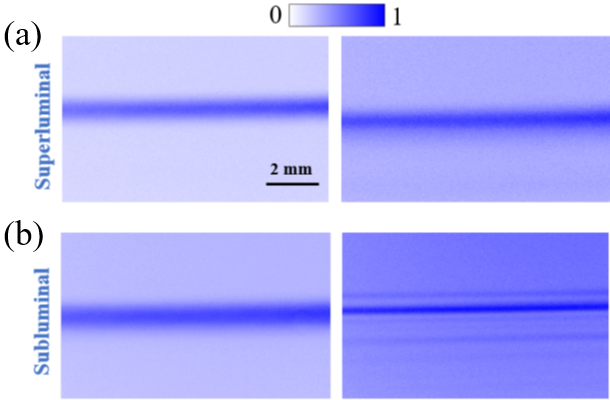


**Figure S3.** Experimental results confirming the second harmonic generation of the superluminal corresponding to *θ* = 45.01° and 45.02° (a) and the subluminal (b) 44.99° and 44.8°, ST wave packets after BBO crystal.

References

1. H. E. Kondakci and A. F. Abouraddy, "Diffraction-free pulsed optical beams via space-time correlations," Opt. Express **24**, 28659 (2016).
2. H. E. Kondakci and A. F. Abouraddy, "Diffraction-free space-time light sheets," Nat. Photonics **11**, 733–740 (2017).
3. B. Bhaduri, M. Yessenov, and A. F. Abouraddy, "Meters-long propagation of diffraction-free space-time light-sheets," Opt. Express **26**, (2018).
